# Adherence to Covid-19 vaccination during the pandemic: the influence of fake news

**DOI:** 10.1590/0034-7167-2023-0284

**Published:** 2024-04-22

**Authors:** Luana Cristina Roberto Borges, Sonia Silva Marcon, Gabrielly Segatto Brito, Miriam Terabe, Nathalia Ivulic Pleutim, Ana Heloisa Mendes, Elen Ferraz Teston

**Affiliations:** I Universidade Federal de Mato Grosso do Sul. Campo Grande, Mato Grosso do Sul, Brazil; II Universidade Estadual de Maringá, Maringá, Paraná, Brazil

**Keywords:** Immunization, Family Health, Fake News, Covid-19, Nursing, Inmunización, Salud de la Familia, Fake News, Covid-19, Enfermería, Imunização, Saúde da Família, Fake News, Covid-19, Enfermagem

## Abstract

**Objectives:**

to understand how fake news has influenced adherence to Covid-19 immunization, from the perspective of health professionals.

**Methods:**

a qualitative, descriptive-exploratory study was conducted in Campo Grande - MS. Twenty nursing professionals working in vaccine rooms or managing immunobiologicals participated through semi-structured interviews. The interviews were audio-recorded, fully transcribed, and subjected to thematic content analysis.

**Results:**

two categories emerged in which the professionals highlighted an increase in vaccine hesitancy among the population, influenced by fake news and denialist actions, which negatively interfered with the population’s trust in vaccines and in the professionals administering them.

**Final Considerations:**

concerns about vaccine safety and denialist actions by authorities and media outlets can contribute to the phenomenon of non-vaccination. The valorization of science, the promotion of educational actions, and raising public awareness about immunization were presented as strategies to increase vaccine coverage

## RESUMO

**Objetivoss:**

apreender como as *fake news* influenciaram na adesão à imunização contra a Covid-19, na perspectiva dos profissionais de saúde.

**Métodos:**

estudo qualitativo, descritivo-exploratório, realizado em Campo Grande – MS. Participaram 20 profissionais de enfermagem atuantes em sala de vacina ou gestão de imunobiológicos, por meio de entrevista semiestruturada. As entrevistas foram audiogravadas, transcritas na íntegra e submetidas à análise de conteúdo, na modalidade temática.

**Resultados:**

emergiram duas categorias nas quais os profissionais destacaram o aumento da hesitação vacinal por parte da população, a influência das *fake news* e de ações negacionistas que interferiram negativamente na confiança da população nas vacinas e nos profissionais que as aplicam.

**Considerações Finais:**

questionamentos em relação à segurança vacinal e ações negacionistas realizadas por autoridades e veículos midiáticos podem gerar o fenômeno da não vacinação. A valorização da ciência, a promoção de ações de educação e a conscientização populacional quanto à imunização foram apresentadas como estratégias para aumento da cobertura vacinal.

## RESUMEN

**Objetivos:**

comprender cómo las *fake news* influyeron en la adhesión a la inmunización contra la Covid-19, desde la perspectiva de los profesionales de la salud.

**Métodos:**

estudio cualitativo, descriptivo-exploratorio, realizado en Campo Grande – MS. Participaron 20 profesionales de enfermería que trabajan en la sala de vacunas o en la gestión de inmunobiológicos, a través de entrevistas semiestructuradas. Las entrevistas fueron grabadas, transcritas en su totalidad y sometidas a análisis de contenido, en la modalidad temática.

**Resultados:**

surgieron dos categorías en las que los profesionales destacaron el aumento de la vacilación vacunal por parte de la población, la influencia de las *fake news* y de acciones negacionistas que interfirieron negativamente en la confianza de la población en las vacunas y en los profesionales que las aplican.

**Consideraciones Finales:**

preguntas sobre la seguridad de la vacunación y acciones negacionistas realizadas por autoridades y medios de comunicación pueden generar el fenómeno de la no vacunación. La valorización de la ciencia, la promoción de acciones educativas y la concienciación de la población sobre la inmunización fueron presentadas como estrategias para aumentar la cobertura vacunal.

## INTRODUCTION

In its historical context, Brazil’s National Immunization Program (NIP) has become a world reference in vaccination coverage, notably for its universal, free, and equal access^(1)^. Despite the NIP’s success in eradicating smallpox, controlling poliomyelitis, measles, mumps, rubella, and in reducing the incidence of vaccine-preventable diseases, questions about the effectiveness and safety of immunization have arisen, sparking debates about individual rights^(2,3)^.

The benefits of immunization are clear, including reducing infant mortality, improving the health and well-being of the population, and reducing the costs of vaccine-preventable diseases for the health system^(3,4)^. Paradoxically, the benefits that vaccines have provided over the years also face challenges, as disease control, due to high vaccination coverage, influences the perception of the risks and benefits of vaccination^(3)^.

Despite evidence of the general benefits of immunization, vaccination coverage in Brazil and in various countries has been threatened by a phenomenon known as vaccine hesitancy - a set of attitudes ranging from reluctance to outright refusal of vaccines, despite the availability of vaccination services^(5)^. This phenomenon has raised concerns about the resurgence of vaccine-preventable diseases, such as the measles outbreak that occurred between 2013 and 2015 in Northeast Brazil^(1)^.

A national ecological study, assessing immunization data from 2013 to 2020, pointed out that vaccination coverage in Brazil in 2020 was the lowest in recent years, with a significant drop compared to 2019. This trend was observed in nine out of the ten vaccines indicated in the NIP schedule for children up to 12 months old, all of which fell below the goals set by the Ministry of Health^(6)^.

Specifically, during the pandemic period, a study using data from the National Immunization Program Information System (SIPNI) on the monthly number of vaccine doses administered to young children in March and April across the country, and data from interviews with parents conducted in August 2020 in 133 large cities in the 27 states, found that the delay and/or loss of vaccination during the period of most intense social distancing was approximately 20%^(7)^. A retrospective analysis also indicated a significant reduction in vaccine administration for children aged 0 to 2 years across Brazilian regions, with the largest decreases in the North (-25.3%), Northeast (-16.8%), and Central-West (-10.2%) regions. For children aged over 2 to 6 years, the biggest drop was observed in the North (-27.2%) and South (-14.0%) regions^(8)^.

However, it is important to emphasize that the decline in children’s vaccination coverage is a phenomenon that had already been observed and cannot, therefore, be attributed exclusively to the pandemic, although it did contribute to exacerbating the rates found. Other factors also deserve attention from health professionals, educators, and managers, and should be the focus of future studies^(6)^.

Regarding the Covid-19 vaccine, in particular, but also applicable to other vaccines, it should be noted that misinformation and the circulation of information from unreliable sources contribute to increased vaccine hesitancy. Digital media have become a conducive environment for the production and circulation of fake news, false messages, or those containing elements intentionally misleading in their content or context, which compromises public health promotion and disease prevention^(5)^. In this context, the question arises: How have fake news influenced the immunization service?

## OBJECTIVES

To understand how fake news influenced adherence to Covid-19 immunization from the perspective of health professionals.

## METHODS

### Ethical Aspects

This study followed the ethical standards of research involving human beings, as per Resolution 466/2012, and was approved by the Research Ethics Committee for Human Beings of the Federal University of Mato Grosso do Sul. All participants expressed their agreement to participate in the study by signing the Informed Consent Form (ICF) in two copies of equal content.

### Type of Study

A qualitative, descriptive-exploratory study. The Consolidated Criteria for Reporting Qualitative Research (COREQ) guidelines were used in the preparation of the research report.

### Methodological Procedures

Potential participants were personally contacted after being referred by the administrative management of each health unit. At this time, they were informed about the objectives of the study and invited to participate. Upon agreement, a date and time for the interview were scheduled according to the participant’s preference/availability, so as not to interfere with the routine of the service.

### Study Setting

The study was conducted in a Health District in the city of Campo Grande/MS.

### Data Source

Nursing professionals involved in immunization at the nine urban Health Units that are part of one of the seven Health Districts of the city were interviewed. This District was chosen for convenience. The predefined inclusion criteria were: being a nursing professional of medium and higher level, working in the vaccine room and/or in the coordination/management of immunobiologicals. Professionals who were on medical leave, maternity leave, external training to the work environment, or on vacation during the data collection period were not included.

### Data Collection and Organization

Data were collected from May to December 2022 through individual semi-structured interviews, conducted in person in a private room at the Health Unit where the professional worked. The interviews were audio-recorded, lasted an average of 30 minutes, and were conducted by the principal researcher. During the interviews, a two-part instrument was used, the first part containing participant characterization questions (gender, age, position, training, employment relationship, experience time in training, experience time in the position). The second part contained a guiding question: “Have you experienced any situation involving fake news or anti-vaccine movement actions? What is your opinion on this?”.

At the end of the interviews, the researcher sought to clarify obscure points and validate the main points/aspects addressed by the participant, so that no interview needed to be repeated. New participants were included until the data started to become repetitive and the research objective had been achieved^(9)^.

### Data Analysis

For treatment, all interviews were transcribed in full and submitted to content analysis, thematic modality, following the pre-established steps that included pre-analysis, material exploration, and results treatment^(10)^. In the pre-analysis, the interviews were organized and floating reading was applied, applying the rules of exhaustiveness, representativeness, homogeneity, and pertinence, in which central ideas were identified. In the exploration of the material, the central ideas were grouped by similarity, forming the following sense nuclei: Denialism and anti-vaccine movement; Obscurantism in governmental actions; The impact of the dissemination of false news; and The influence of the media on vaccine adherence. After in-depth analysis and guided by the objectives, a thematic category originated. In the treatment stage, with significant and faithful results, it was then possible to propose inferences and advance interpretations regarding the results found^(7)^, considering the predetermined objective and the premises of the NIP.

## RESULTS

Twenty nursing professionals, aged between 25 and 53 years, participated in the study, of whom 16 were female; there were 13 nurses, three of whom worked in management. Of the seven nursing technicians, two held a higher education degree, and of the 13 nurses, four were specialists in family/public health; six in other health areas, and three held master’s degrees. Their professional experience ranged from 18 to 240 months.

### Influence of Fake News on Vaccine Credibility During the Covid-19 Pandemic

The participants highlighted the rise of denialism and the intense spread of fake news during the Covid-19 pandemic, mainly related to vaccines manufactured and used during that period. Furthermore, they pointed out various repercussions in direct user service and in their interpersonal relationships.

According to the participants, fake news had an impact on the replication of incorrect information by users regarding the safety, efficacy, and adverse events of Covid-19 vaccination.

[...] *Not only for Covid but also for influenza and various other vaccines, people believe they are designed to kill the elderly, that they are ineffective, and that they cause genetic mutations.* [...] *We are witnessing the resurgence of diseases that had been eradicated due to these anti-vaccine movements, fueled by the belief that if it’s from the SUS* [public health system]*, it’s not good, even though it’s from the same manufacturer as the private clinics.* (EnfA3)

*“I’m only going to take it because I need to travel, and they won’t let me board if I don’t,” “I won’t take it because they said this vaccine causes sterility,” “I prefer this vaccine over that one because that one is killing people,” “someone took it, had a reaction, and died” was what I heard most often.* (TEnfG1)

In the general context, they negatively influenced public health by fostering vaccine refusal.

*I remember a man who came in one day and said, “I came here to tell you that I’m not going to take the vaccine, you understand? Because I already know what the vaccine does, I read about it in a WhatsApp group, and I just came to tell you that I’m not taking it”.* (EnfA2)

*I’ve seen many patients who come into the office and say they haven’t been vaccinated and aren’t going to be, especially parents now who don’t want to vaccinate their children because they themselves didn’t get vaccinated. I think this is a significant regression; people didn’t use to question what was in the vaccine. For example, the meningitis vaccine, one of the side effects it can cause is meningitis itself, so* [laughs] *it’s complicated, right?* (EnfA6)

Furthermore, this led to an increase in users’ distrust of certain vaccines, insistence on choosing specific immunizers, and attempts at fraud related to vaccination records.

[...] *Regarding patients, it was about wanting to choose their immunizer, so that was the problem. Often the patient would come in, and we had a large stock of Coronavac, which was the one we were administering at the time, then the patient would say, “I want to take Pfizer”.* (EnfA5)

*I’ve seen people trying, asking, and I’ve also heard colleagues in other units recount similar stories* [...]*, with some people asking, for example, for an employee to make a fake vaccination certificate. In our unit, there’s a more elite segment of the population that sought us out for a specific vaccine because they were traveling abroad and some countries only accepted certain vaccines. Sometimes they were already vaccinated but with a different vaccine and asked us to make a different certificate. I’ve seen these attempts to get a certificate without being vaccinated, and the opposite also happened.* (EnfA10)

At times, professionals’ opposition to fake news was perceived as a factor that strained their interpersonal relationships, whether familial or professional.

[...] *There were several reasons for conflicts within families; I even left family groups and argued with relatives over these fake news. There was always one side defending the fake news and another side trying to debunk it, showing it was a lie.* (EnfA1)

*The elderly are the ones who bring the most fake news; they chat in line, and when they come in, they have 80 pieces of news to tell me about the vaccine. Without patience, you can’t vaccinate; he leaves with doubts, and it takes on a much larger proportion. These things affect our work, and we need a lot of patience to explain, especially to parents who say, “no, I only want the routine ones, I don’t want Covid,” it’s a total anti-vaccine movement, “because my child is not a guinea pig”.* (TEnfA3)

Obscurantist governmental actions were also cited as aggravating factors for the spread of denialism and anti-vaccine movements. This was due to the then president of the country and authorities from the Ministry of Health not only publicly opposing health recommendations but also discouraging such measures. Additionally, they promoted treatments that were proven ineffective and harmful to health.

[...] *What hits me hardest now is the political issue. Considering that the federal government was notably against the vaccine, against science, with speeches contrary to this. As the leader of a government, of a country with 200 million inhabitants, a discourse like this against the vaccine would certainly impact not only the Covid vaccine but other vaccines.* (EnfA1)

*I think it’s terrible, especially because we don’t have government support. It made things even worse because science said one thing, and other people said something else. It interfered with all the work we were doing. I remember the vaccine revolt, a historic and terrible moment.* (EnfA2)

[...] *I believe it’s very much linked to the political climate we live in, the reinforcements from politicians on this theme, who are influential people and, whether they want to or not, they influence most people who believe in them. So I think this political bias, or even famous people in different areas* [artists, players, singers...] *make people believe, fanaticism is also very much linked to this, and it’s a big challenge.* (EnfA4)

The media’s role in disseminating false information was also identified as influencing population adherence to the vaccine and the credibility of professionals involved in immunization

*The media also had this* [misreporting of information]*. We had so much national impact from fake news or conflicting information just like within the municipality, within groups. Just as it helped, if you weren’t critical, just received and passed on, it hurt a lot.* (EnfG1)

Moreover, media coverage of atypical cases, such as frauds, errors, and losses of vaccines, contributed to users’ distrust at the time of immunization. Consequently, it became necessary to implement protocols and processes for vaccine administration that would contribute to the transparency of the process in order to minimize the population’s insecurity.

*Like there were other professionals, in other cities, who acted in bad faith by not administering the vaccine, and this misinformation spread. It was embarrassing for us vaccinators, with everyone wanting to check the syringe, wanting to see if the vaccine was really administered. There were even accusations like, “you didn’t do it,” “where did it go?”, “what happened?”. This was very challenging.* (TenfA1)

[...] *You can’t administer the vaccine without showing it to the patient, so we always showed the vaccine, the color, the syringe, the bottle, explained how many ml, showed the empty syringe after administering, and it became a routine that everyone adopted because it helped; if you didn’t show* [...] *that issue of fake news.* (TEnfA3)

Regarding the relationship between professionals and service users, the sometimes inappropriate image portrayed by the media during the most intense period of immunization was also highlighted as an influence on vaccine denialism.

[...] *There was a day when a report came at 2 in the afternoon, and we had 130 people to attend, with only eight technicians; it’s not humanly possible. There was nothing to do, how to open another room because we don’t have a thermal box, we don’t have the material, we have vaccines but no materials, no other room, with other appointments happening. So the report was here in front of the unit criticizing, calling, saying we were denying, and the health professional was portrayed as the villain.* (TEnfA3)

*We suffered harassment because of Covid. The media itself turned the public against the vaccinators, saying that the vaccinators were vaccinating incorrectly* [...]*. Instead of showing the population that it is viable, that the technicians are well-prepared, they wanted to portray an unprepared team.* (TEnfA2)

In conclusion, the importance of disseminating scientific knowledge and valuing educational measures to mitigate the effects caused by fake news in health services was emphasized.

*There are many people who value the health professional, so there are people who come not even to provoke or question the vaccine, but to know our professional opinion about it, “ah, I saw something on the internet, but I wanted to hear it from you,” and then it’s an opportunity you have to reinforce what is scientifically based.* (EnfA8)

*I think we really have to equip ourselves with true information, actual facts, science. So if you Google something and it generates doubt, it’s very fitting to go to a reliable source and verify what is true before spreading false information. You could be harming someone else who might have been vaccinated, and because of that information, you created doubt in their mind, and they chose not to vaccinate. And this information can spread, because word of mouth spreads a lot. So I think it’s a cascade of bad information that is disseminated among the population.* (EnfA11)

In summary, it was evident that the rise of denialism, in conjunction with the spread of false news, reflected in users’ attitudes towards vaccines. Topics related to vaccine adherence and reliability, and the valuing of science and health services, can be strengthened through educational practices in health.

## DISCUSSION

The findings of this study have led to the formulation of hypotheses that may have contributed to the phenomenon of vaccine hesitancy regarding the Covid-19 vaccine. For example, the influence of fake news on the immunization service was highlighted, especially in terms of reducing the population’s trust in vaccines and the professionals administering them. Participants also emphasized the excessive questioning about vaccine safety, as well as denialist actions by public figures (government officials, artists, athletes, influencers), official health agencies of the country, and media outlets, which increased the complexity of the health crisis experienced during the Covid-19 pandemic. Consequently, participants pointed out the need for valuing science and promoting educational and awareness-raising actions regarding immunization.

It is important to note that the term “fake news” is used to describe the harmful practice of producing and disseminating false news on a large scale, with the intention of intentionally distorting facts^(11)^. During the Covid-19 pandemic, there was a massive increase in the spread of false information in Brazil, especially related to the coronavirus, vaccines, and public health. This could be related to the hyperconnectivity of the population, most of whom are unable to differentiate between false and true news and have difficulty verifying the veracity of the information they share^(12)^.

The increased access to and use of social media and messaging apps as primary sources of information also facilitate the spread of fake news. Young people and individuals with low educational levels are more prone to trust the information available on these sources^(13)^. This occurs due to skepticism and mistrust of traditional sources of information, such as media outlets, academic, scientific, and political institutions. Thus, so-called “scientific” information does not reach the population adequately^(14)^.

This resistance to reliable sources may be related to difficulties in accessing scientific content, understanding academic language, and the way the content is presented. There is a frequently observed pattern in fake news that includes novelty, objectivity, appealing and emotional language, sensationalist headlines that generate indignation and consequent attention from the reader, and which most likely reinforce pre-existing confirmation biases^(15,16)^.

It is important to highlight that social networks, messaging apps, and media dissemination channels are not designed for disinformation purposes. However, conspiracy theories, fantasies, and sensationalism generate audience and engagement, which are converted into credibility and financial profit, valuable items in the contemporary context^(12)^. Thus, the spread of false news is a complex sociocultural phenomenon, influenced by various behavioral and social factors that affect the perception and judgment of truthfulness. This contemporary problem is not limited to political and social spheres but also represents a risk to public health^(12)^.

In this context, fake news as a mechanism of manipulation endangers the achievements and advances made over the last 50 years by the National Immunization Program in relation to vaccine-preventable diseases. A study on the acceptance, trust, hesitation, and refusal of vaccination against Covid-19 conducted with 1,599 people in Canada indicated that 88.9% of participants generally accept most or all recommended vaccines, while 18.2% indicated vaccine refusal for the Covid-19 vaccine^(17)^. Meanwhile, a survey conducted with 229,242 people in Korea estimated that only 3.9% of the adult Korean population refused the Covid-19 vaccine^(18)^.

It is important to highlight that the World Health Organization (WHO) has listed vaccine hesitancy as one of the top ten threats to health that need to be addressed, especially since it endangers the lives of millions of people who benefit from immunization^(19)^. In light of this, information that feeds irrational fear in the population requires adequate oversight to establish norms, guidelines, and transparency mechanisms to ensure safety, freedom of expression, communication, and the manifestation of thought without violating constitutional principles, rights, and guarantees.

Regarding the attitude of parents towards their children’s vaccination status, a national study conducted in Canada with 6,519 parents of 2-year-old children showed that 16.8% of them had refused a vaccine in the past, with the most commonly refused vaccines being influenza (73%), rotavirus (13%), and varicella (9%). About 12.8% were hesitant to vaccinate their children, especially against influenza (34%), measles/mumps/rubella (21%), and varicella (19%)^(20)^. In Turkey, a survey of 396 parents of adolescents aged 12 to 18 years indicated that 41.7% refused to vaccinate their children against Covid-19^(21)^.

In the Brazilian scenario, the estimated vaccination coverage for Covid-19 is 85% of the population vaccinated with the first dose, 80% with the second dose, and 50% with the booster dose^(22)^. Considering the vaccination coverage goals of 90% proposed by the Ministry of Health, and the significant drops in immunization rates over the years, it is noteworthy how these factors act on the impossibility of achieving the so-called herd immunity that occurs when a significant percentage of individuals in a population acquires immunity to a disease^(23,24)^. Although refusal or hesitancy remains a minority in the population, it is important that vaccination incentive campaigns be strengthened, as the risks related to this phenomenon tend to harm the entire population, making it more susceptible to contracting and transmitting diseases that were previously controlled.

Furthermore, the results highlighted the importance of the stance of public figures representative of the population in the face of public calamity situations, exercising their social function for democracy. At times, the posture and conduct adopted by public figures and opinion leaders can constitute a support point for denialism, which makes it difficult to reach vaccination goals in the Brazilian population and contributes to distrust of the health service and the performance of the professionals working in it^(11,12)^.

Pertaining to this, actions of polarization around vaccination were fostered, along with the encouragement of unnecessary politicization, such as in the resistance to acquiring CoronaVac, produced in collaboration between the Butantan Institute and Sinovac. This fact led the population to request a choice of vaccine brand at the time of immunization, causing disruptions for vaccinators^(12)^.

In this study, the role of the media was sometimes also pointed out as having a negative influence on population adherence to immunization. During the Covid-19 pandemic, news was disseminated that emphasized situations of loss, fraud, illegal sales of vaccines, and immunization errors. According to the guidelines of the National Immunization Program (NIP), failures in vaccine administration can result in reduced or absent effects, as well as adverse events post-immunization. These errors also have negative implications for the population, leading to interruptions in vaccination protocols, a decrease in vaccination coverage rates, and threats to the control of diseases that could be prevented, generating both direct and indirect costs for health services.

In a study conducted in a Brazilian state that evaluated 3,829 notifications of immunization errors in the database of the Surveillance System of Adverse Events Post-Vaccination (SI-EAPV), it was observed that children under 1 year of age were the most affected (39.1%), and the intramuscular route was responsible for 29.4% of the errors. The most frequent error was administering a vaccine outside the recommended age (37.7%)^(25)^. It is important to note, however, that despite the work process in public institutions being governed by strict norms and guidelines prioritizing the well-being of the population, it is not immune to errors. Therefore, it is important that any failures be reported, analyzed, and followed up individually, and subsequently discussed among the health team to identify possible flaws in the work process and to strengthen measures to prevent Supposedly Attributable Events to Vaccination or Immunization (SAEVI).

It is emphasized that, in times of health crisis, ensuring the well-being of society is the primary goal. In this way, health professionals, especially nurses who worked directly in vaccination rooms, assumed a central role in combating the spread of misinformation. It is reiterated that the positioning of professionals in the face of fake news, due to their scientific knowledge and ethical commitment, becomes essential for the effectiveness of the NIP^(26)^.

Health education is a fundamental pillar of the health promotion strategy, through the implementation of disease prevention actions. When executed efficiently, it allows the population to become emancipated, democratize knowledge, and participate actively, increasing the adoption of healthy habits with a direct impact on individual and community health^(27)^. In the current health panorama, it is indisputable that the nurse plays a significant role in the development of educational actions, regardless of the space they occupy in the Health Care Network. This is because they present themselves as a facilitator of the teaching-learning process, capable of disseminating scientific knowledge, contributing to the validation of science. Caring and educating are, in fact, complementary attributes that cannot be dissociated in the nursing work process.

### Study limitations

This research has limitations that should be considered. Related to the study design, it is noteworthy that it involved interviews with a specific group of professionals, which limits the precision of the impact of various aspects on the phenomenon of vaccine hesitancy. Additionally, it is important to note that the topic addressed is influenced by sociocultural, political, and partisan factors. During the interviews, moments of silence, laughter, jokes, and resistance from some participants were observed. To mitigate these issues, the researcher carefully reformulated and directed the questions to ensure that the study’s objective could be achieved.

### Contributions to the Field of Nursing

The results of the study reiterate the indispensable role of the nurse in rescuing and maintaining the importance of the prevention of vaccine-preventable diseases and the role of the National Immunization Program (NIP) in their daily practice. As they work directly in immunization, the nursing team continuously needs to correct or redirect service users, providing evidence-based information, with the aim of contributing to decision-making in the health-disease process and adherence to vaccination. With a view to promoting quality health care, it is essential that institutions have access to the most up-to-date information and train their staff to deal with the diversity of situations and identify fake news. Constant training is indispensable to keep the entire team prepared and capable of facing the challenges that the health service routine can present, with prioritizing this update being synonymous with a commitment to the excellence of care.

## FINAL CONSIDERATIONS

We offer support for discussions and reflections by professionals and managers regarding the need for strategic actions to address vaccine hesitancy in the context of Primary Health Care, supporting the adoption of educational measures aimed at empowering health workers to combat misinformation through health education, as well as strengthening the role of nursing in valuing the NIP. It also enables reflection on the role of management in supporting and valuing professionals who, through their actions, are on the front line in facing actions aimed at weakening the Unified Health System (SUS) and its health policies.
